# Future Time Perspective and the Achievement of Optimal Best: Reflections, Conceptualizations, and Future Directions for Development

**DOI:** 10.3389/fpsyg.2020.01037

**Published:** 2020-06-03

**Authors:** Huy P. Phan, Bing H. Ngu, Kelvin McQueen

**Affiliations:** School of Education, University of New England, Armidale, NSW, Australia

**Keywords:** time, future time orientation, extended timespan, optimal best, optimization, positive psychology

## Abstract

*Time* is an interesting concept. For some cultural groups, time is an entity that exists only in the here and now, whereas for others it can be linear, emphasizing a person’s past, present, and future. Many of us, while living in the “present moment,” may also anticipate and project future goals, dreams, hopes, and ambitions. Indeed, from a positive point of view, future orientations are healthy and may direct one’s focus, instill motivation and persistence, and mobilize the expenditure of effort. Existing research has provided empirical evidence to support the promotion and encouragement of a positive *future time orientation*. From an educational point of view, the study of time may be useful for calculating achievement, given that a student may use future time orientation to guide and direct his/her academic and/or non-academic future. One notable question for consideration, in this case, relates to the *importance of timespan* – that is, how far into the future should one project? There may be a significant difference between, say, a timespan that scopes a 6-month period as opposed to a timespan that scopes a 2-year period. By the same token, over the past few years we have delved into an interesting line of inquiry, namely, the *nature of optimal best* – for example, what facilitates and/or causes a person to achieve an optimal level of best practice in particular subject matter? Our theory of human optimization, consolidated and recently published in *Frontiers in Psychology*, provides an in-depth theoretical account of an underlying process, which we postulate could help explain the achievement of optimal best. Optimization, in this case, is intimately linked to a person’s achievement of optimal best. We rationalize that within the context of academic learning, cognitive complexity of particular subject matter could serve as an important source of motivation in the anticipation and projection a student’s extended future timespan. In this analysis, the extremely complex nature of a learning task or a suite of tasks may compel a student to consider a longer future timespan for successful completion. We also argue, in contrast, that the specific duration of a future timespan (for e.g., 6 months vs. 2 years) could play a significant role in the successful optimization of a student’s state of cognitive functioning.

## Introduction

*Time* is an interesting and mysterious concept. For Albert Einstein, space and time are merged inextricably into a four-dimensional space-time continuum. Buddhist philosophy holds the philosophical position that there is no past and there is no future; only the present moment. This belief means that we should not ponder past events, nor consider and plan for the future. Rather, in tandem with Eastern understandings of mindfulness and meditative practice (Nyanaponika, 1972; Master, 2010) everything is here and now. *We live in the present moment*. The authors appreciate this philosophical premise, but contend that our own personal beliefs, research development, and professional experiences differ from Buddhist philosophy and we acknowledge and recognize the importance for modern physics of time being linear. But in this study, the personal experience of time is the essence for consideration; like when people ask which nation will next win the next FIFA World Cup; when are we likely to experience another financial crisis; what will the next iPhone look like; and so on? When people pose questions like these, for example, then they are rejecting a viewpoint of life being lived in just the present moment.

*Time perspective*, commonly known as TP, is an interesting theoretical concept that has received considerable research interest over the years (e.g., Zimbardo and Boyd, 2008; Milfont et al., 2012; Janeiro et al., 2017). Consideration of time perspective, from our synthesis and review of the literature, can explain in part the relevance and personal significance of life experiences. Time perspective, in this case, considers time a non-singular entity (Zimbardo and Boyd, 1999) through which a person self-reflects upon his/her past experiences to shape their present moment, which in turn informs his/her future actions. The future, in this sense, indicates different possible outcomes, both positive and negative, depending on personal estimations of past and present life experiences. Flowing from this, the authors acknowledge that time, in terms of its continuity, has relevance, significance, and applicability in relation to the achievement of both educational and non-educational outcomes. A student’s previous negative experiences in mathematics, for example, may give rise to his/her present state of anxiety when learning this academic subject, which would also be likely to deter him/her from choosing a mathematics-related career in future. In a similar vein, an adolescent’s positive feelings when working with senior citizens may motivate him/her in future to enroll in a social work degree.

With reference to the study of TP, *future time orientation* is an interesting timepoint for analysis and examination. As individuals, both in academic and non-academic spheres, we are always curious and interested to know what the future holds. Will we achieve exceptional grades; will we have enough savings for retirement? In the context of schooling, in the case under consideration here and according to several commentators, future time orientation plays a meaningful role in motivating students to seek new frontiers and to strive for successful accomplishments (Lens et al., 2002; Simons et al., 2004a, b). With this in mind, one interesting element of enquiry is the *specific extended timespan into the future* that would be considered optimal. What is the most adequate timespan into the future that would yield productive and enriching outcomes for a person? There are individual variations in perceptions of future time, for example: a 2-week timespan into the future for a 4-year-old child is quite lengthy as they anticipate their birthday present (e.g., “What will I get for my birthday?”), as opposed to a 5-year timespan into the future for a 17-year-old teenager who wishes to become a medical doctor. From the motivational literature, anticipating and setting forth a specific timespan into the future is a valuable consideration. How briefly or deeply into the future we anticipate appears to influence our internal motivational state. It becomes motivational and proactive for a person to anticipate and set forth an extended timespan into the future for attaining positive yields.

One focus of enquiry to consider is the establishment of different pathways and means for encouraging the contemplation and projection of specific timespans into the future. At the same time, of course, there is the need of directing a person’s focus toward actually seeking achievement of future ambitions. In considering both these needs, a possible line for development is motivating people to achieve *optimal best* (Martin, 2006; Liem et al., 2012; Phan and Ngu, 2019a). Optimal best, that is, the attempted maximization of a person’s state of functioning, may enhance the active processes of human agency. We contend that striving to achieve optimal best in school subjects, for instance, could assist a person to project a specific and definitive timespan into the future. In a similar vein yet conversely, a developing a specific timespan for future achievement could help galvanize a person’s focus and motivation to achieve optimal best.

We acknowledge that to date very little, if any, focus in the relevant literature has been given to considering the relationship between TP and the achievement of optimal best. Do the two theoretical concepts relate to each other and/or to what extent do these two theoretical concepts explain proactive human behaviors? This line of inquiry, theoretically and conceptually, is innovative for its positive nature, reflecting our recent research into the study of optimal best (e.g., Phan and Ngu, 2017a; Phan et al., 2019a,b, c). As a working hypothesis, we could argue that optimal best is necessarily reliant on a point of reference structured into the future. In other words, unlike a person’s historical optimal best, which is past, optimal best as a positive outcome requires time for achievement, i.e., achievement in the future. In that case and as one consideration, how much time is needed to achieve optimal best may depend on the cognitive complexity of the subject matter. Furthermore, having an extended timespan into the future is, perhaps, a necessity for the achievement of optimal best. Overall then, we contend that a focus on TP within the context of optimal best is significant, contributing to advance the research into the optimization of optimal best.

## The Importance of Time

*Time*, as we briefly described, is an interesting concept that has implications for society and individuals. Why does a local government need to plan ahead in terms of policy development? Why is a secondary school student being asked to reflect on his/her previous academic performance in mathematics? Why does a footballer have to focus on her/his current state of physical functioning? These questions are significant for this study since they place emphasis on a linear trajectory and, more importantly, suggest potential interrelations between past, present, and future events. Some individuals, of course, may simply fixate upon past situations and experiences, whereas others may choose to ponder their future. This diversity is interesting as it acknowledges that to a large extent our lives are intricately linked to the nature of time. Erikson’s (1968) *psychosocial theory of personality* with its eight stages, for example, places emphasis on a linear progression from one level to the next. For Erikson (1968) completion and success at a particular stage form the basis for continuing onto the next stage. This theory emphasizes the importance of time as a linear trajectory, as a pathway and pattern of development, where past experiences contribute to influencing the present moment and, likewise, the future.

It is interesting, however, that other viewpoints do not necessarily concur with the notion of the continuation of time. Let us delve into this positioning a bit. Our research development in *mindfulness* from an Eastern perspective (Hanh, 1976; Yeshe and Rinpoche, 1976; Goldstein and Kornfield, 1987; Loden, 1996; Kabat-Zinn, 2015) has offered a different viewpoint about the concept of time. That viewpoint is that time is a non-linear entity. Time is not thought of as continuous and there is no associated discourse of temporal linearity. Mindfulness, according to Taiwanese colleagues known to the authors who are practicing Buddhist nuns and monks, is concerned solely with the *present moment* – everything is here and now. Buddhist scholars, for example, believe that the past does not matter and, likewise, we do not plan for the future since we do not know what it holds. Life in the present moment is straightforward and has few complications. Thus, the personal experience of the present moment is said to reflect a state of *mindfulness*; a state of clearness, calmness, and enlightenment. This philosophical position, derived from Buddhist sutras (Yeshe and Rinpoche, 1976), places emphasis on concentrating on the essence of the context as the “here and now.”

Indeed, we acknowledge that “situating” life in the present moment appears to be “simple” and straightforward. Why worry and stress about the future when one does not know what this future may hold? Similarly, why reflect upon and ponder the past when it is impossible to amend events, situations, and/or experiences? Buddhist teaching, in this sense, is related to the notion of a person living and leading a simple, healthy, and happy life. We acknowledge, however, that this viewpoint is largely incompatible with Western theorizations and understandings of temporal linearity. For example, Nuttin’s (1964) article, entitled *“The future time perspective in human motivation and learning,”* provides an interesting reference to time: “A simple analysis of human behaviors calls attention to the fact that man [and woman], in his [/her] dealing with a given situation, is usually directed toward something which is not yet there, something which is still to come, something different, even something new…, are all oriented toward something ahead, something that they are looking for: their behavior is ‘future bound’….” (p. 60). This conceptualizing of time emphasizes the importance of a linear trajectory that connects a person’s past experiences, that is, his/her current state of functioning, into considerations about the future. Moreover, Nuttin (1964) proposes a mysterious and unknown nature to future specific timepoints.

We do not discount the possibility that everyone could, if so inclined, perceive time as something “singular” (i.e., limiting itself to the present moment). The benefit of such a viewpoint is its straightforwardness – that is, it encourages a person to free himself/herself from the complexities and perplexities of life and, from this, perhaps to live “a happy, healthy life in the present moment.” Having said this, however, we acknowledge that in the modern world with its particular type of economic development and perpetuation of certain types of social arrangements, and the associated expectations of personal growth, require in many circumstances the rejection of this viewpoint and the acceptance, in contrast, of a viewpoint that time is continuous. The associated psychological state is that people expect to reflect, envisage, and anticipate different timepoints in their lifetimes. The study of the psychology of time by different researchers (e.g., Lewin, 1942; Nuttin, 1964; Mehta et al., 1972; Zimbardo and Boyd, 1999, 2008) is insightful as this line of inquiry makes a concerted effort to explain the differing patterns in cognition, motivation, and behavior of individuals in both educational and non-educational contexts. Mehta et al.’s (1972) writing provides a brief historical summary of other writers’ understanding of time (e.g., Israeli, 1934; Frank, 1939; Lewin, 1942; LeShan, 1952; Fraisse, 1963; Nuttin, 1964). So, to propose a technical definition of what is time perspective, commonly known as TP, we can turn to Zimbardo et al. (1997) who define TP as “the manner in which individuals, and cultures, partition the flow of human experience into the distinct temporal categories of past, present, and future. The boundaries, extension, salience, and utilization of any of these categories may vary considerably as a function of learned preferences that become stabilized into a functional cognitive style, and also as a consequence of situational, structural, and task demands” (Zimbardo et al., 1997). Extending this, TP perhaps takes on its most important aspect, from a layperson’s point of view, as the ability to anticipate future situations and events by reflecting on his/her past experiences (Lennings et al., 1998). This common-sense understanding proposes that a person’s life experience and growth are “sequenced” in a linear trajectory. A student’s past experience of racism at school, for example, is likely to shape his/her current thinking and behavior, which may then link with deliberations about future actions (e.g., avoid attending school). In contrast, likewise, a student’s current enjoyment of Industrial Arts (e.g., woodwork) may determine his/her choice of a career choice later on.

### The Nature of Future Time Perspective (FTP)

One notable timepoint that is of interest for discussion is the unknown future. Future time perspective, commonly known as FTP, is defined as “the timing and ordering of personalized future events” (Wallace, 1956). Nevertheless, there are differing definitions of FTP. For example: “FTP is the degree to which and the way in which the chronological future is integrated into the present life-space of an individual through motivational goal-setting processes” (Husman and Lens, 1999); or “the totality of the individual’s views of his psychological future and psychological past existing at a given time” (Lewin, 1951); or “a general concern for future events” (Kastenbaum, 1961); or “a general capacity to anticipate, shed light on and structure the future” (Gjesme, 1983); or “the present anticipation of future goals” (Simons et al., 2004b). These differing definitions, according to Seijts (1998), emphasize the complex nature of FTP. A point of commonality in these definitions is that FTP is related to and espouses the importance of a person’s current envisaging of future events, situations, tasks, etc., in his/her present timespace – for example, while writing up this manuscript, we envisage and anticipate its completion in a few weeks from now.

Seijts (1998) definitional overview is interesting for its description of five major facets of FTP: (i) *extension*, which is concerned with the length of the future timespan that a person conceptualizes; (ii) *coherence* is the degree of organization of envisaged events in the future timespan; (iii) *density* is defined as the number of events that are expected in a person’s future – that is, his/her goals, hopes, fears, and wishes; (iv) *directionality*, which is the extent to which a person perceives himself/herself to be moving forward from the present moment into the future; and (v) *affectivity* relates to the extent to which a person feels gratified by anticipated events. These five facets, in totality, play a central role in the operational functioning of FTP. For example, as existing research has shown, the extension of a person’s FTP is closely related to his/her cognition and motivational patterns (Simons et al., 2004a,b). Indeed, developments in research over the past seven decades have produced clear and consistent evidence highlighting the significant impact of FTP on different types of adaptive outcomes (e.g., Zimbardo et al., 1997; de Bilde et al., 2011; Lens et al., 2012; Phan, 2015; Husman et al., 2016; Janeiro et al., 2017).

From a motivational perspective, FTP may operate to encourage a person to be purposive and self-regulated and to flourish in the course of academic learning and schooling (Zimbardo and Boyd, 1999; Barber et al., 2009; Janeiro et al., 2017). It is important, in this analysis, for a person to consider a specific future timespan in terms of envisaging different types of endeavors for accomplishment. This notion of varying extensions of future timespan may serve as a significant source of motivation (Simons et al., 2004a). In general, we may have either short or long (or deep) extended timespans into the future (Simons et al., 2004b). This variation of FTP (e.g., say, 4 weeks into the future vs. 2 years into the future), as the literature has shown, is intricately linked to a person’s state of motivation, cognition, commitment, and behavior. Academically, for example, two fourth-year undergraduate students may aspire toward obtaining Ph.D. degrees in 5 years’ time. One student, Chou, has a long FTP, whereas David, his best friend, has a short FTP. Consequently, the psychological distance toward this future goal of obtaining a Ph.D. is experienced differently by both students – Chou, in this case, is likely to perceive having a Ph.D. as being closer in time than David because of the latter’s short FTP. For David, the same future goal may not be part of his life space. Obviously, there is a negative correlation between an individual’s FTP extension and his/her perceived psychological distance toward the self-set future goal (in this case, obtaining a Ph.D. degree) (Moreas and Lens, 1991). What is of interest also, however, is that the length of a person’s FTP does not affect his/her perceived psychological distance from the goal when this goal is set in the very near or very far future. In this sense, tomorrow or Friday is very near for all of us regardless of the various lengths of our FTPs, whereas 20 or 25 years from now may be chronologically too far away to matter.

Simons et al.’s (2004b) overview of FTP highlights an interesting theoretical aspect from De Volder and Lens’ (1982) research – namely, the distinction between the *cognitive component* and the *dynamic component* in FTP. Consider the aforementioned example of Chou and his best friend, David. Chou, with his extended FTP, is likely to perceive his present behavior as being more instrumental as this would help him achieve a broad range of both immediate and future goals. This is the cognitive component. At the same time, Chou also values his present task-engagement more strongly because the anticipated value of the future goal of obtaining a Ph.D. is higher. This is the dynamic component. David’s case, by contrast, may be somewhat different. Because of his relatively short FTP, David perhaps does not anticipate more-distant future goals and, consequently, his present actions are perceived as less instrumental and/or containing less utility.

In sum, from the forgoing discussion, extended timespans into the future play a significant role in helping to determine and explain individuals’ motivational beliefs and patterns in learning, cognition, and behavior. In this analysis, the study of FTP is not simply limited to the notion of “setting forth future goals for accomplishment” This line of reasoning, we contend, is too narrow and does not reflect the complex nature of a future timespan. A timespan into the future, as we described it, may be relatively short and indicate a simple goal or focus for consideration – for example, what will a person get for her birthday next week? We would contend that it is instead of more value, both in academic and non-academic pursuits, for individuals to consider extended timespans. Individuals with extended FTPs, the literature strongly suggests and we concur, are more likely to be motivated to engage closely with their learning (De Volder and Lens, 1982). These individuals (the example of Chou) perceive their present behavior as being more instrumental in achieving immediate and future goals. Likewise, individuals with extended FTPs value their present task-engagement more strongly because the anticipated value of the future goal is higher (Simons et al., 2004b). The pervasive question of course, arising from this analysis, is the appropriate and optimal extension of FTP.

We contend that it is noteworthy for educators and researchers to develop and explore pathways, means, and/or opportunities that could encourage and foster a healthy extension of FTP. This line of inquiry reflects the importance and benefits of a purposive extended timespan into the future. We do not consider there to be much value in having a short future timespan (e.g., preparing for the class quiz scheduled for next Friday) as this “briefness” would not have any meaningful impact on an individual’s growth. From the perspective of formal education, we believe that it is an important feat for a student to contemplate the different types of positive outcomes that could arise from him/her having an extended FTP. As noted previously, it is probably more significant to focus on and think about university in 3 years’ time than to think about the final exam at the end of this year. Contemplating something that is “deep” into the future is, we believe in concordance with the literature, motivational and may operate to guide and direct a person to plan and strive for successive accomplishments. Career advice and university open days in Australia, for example, play a central role in helping secondary school students think about their futures post-secondary school. Aside from this opportunity, what else is available to help students envisage an extended timespans into the future? Of course, interlocutors could have students write down and discuss both their short-term and long-term future plans. It is also plausible to use vicarious information (Bandura, 1986, 1997), such as observation and role modeling, to facilitate in-depth understanding and appreciation of extended FTPs. Watching and/or observing a credible model who is timely in decision making and who is successful with his/her future endeavors (an ideal example may be someone saying to students the following: “I was successful by the age of 24 to achieve […]. I planned this when I was 18…”) may, in this analysis, provide vicarious information that the individual, too, may succeed with his/her future.

Our research interest, in this case, relates to the development of a conceptualization that could inform and facilitate the extension of an appropriate timespan into the future. Specifically, our focus of inquiry makes attempts to consider the theory of optimization (Phan and Ngu, 2017a, 2019b; Phan et al., 2017, 2019c) as a means by which one could extend his/her FTP. This consideration, we contend, is significant as it places emphasis on the tenet of optimal achievement best (Phan and Ngu, 2017a; Phan et al., 2017, 2019c) and relating this optimal best to a person’s extended FTP. One particular aspect that we rationalize and argue for is that *a deep timespan into the future is more advantageous than a short timespan*. We incorporate theoretical understandings of optimal achievement best to support this proposition – that is, for example, the cognitive complexity of optimal achievement best would closely associate with a person’s specific timespan into the future. An extremely complex level of optimal achievement best would extend a person’s timespan (say, 6 months), whereas a low complex level would shorten his/her timespan (say, 2 weeks).

## Bidirectional Associations Between FTP and Optimal Best

We rationalize the potential bi-directional interrelationship between FTP and the nature of optimal achievement best. This conceptualization, which we explore in detail in this section and subsequent sections, is innovative as it draws attention to two lines of research: (i) the stipulation of an extension in FTP in order to achieve optimal best; and (ii) the complexity of optimal best, which, we have asserted, closely aligns with an extension in FTP. Our conceptualization, in general, proposes the promotion and fostering of an extended FTP and the setting of an optimal best that has high cognitive complexity.

### The Framework of Achievement Bests

**FIGURE 1:**
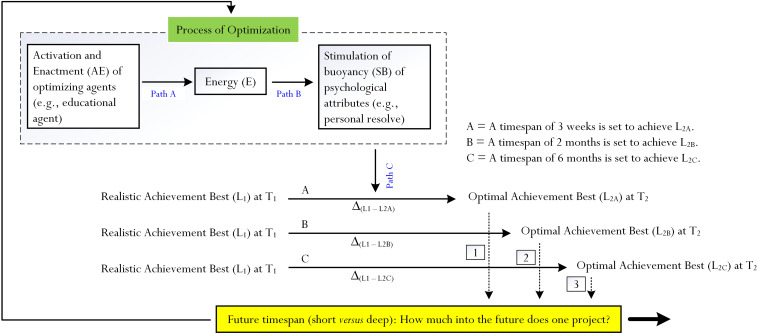
Conceptual model for consideration. Note: this conceptualization is derived from Phan et al.’s (2019c) theoretical model of optimization. According to this theoretical model of optimization, the activation and enactment of different types of optimizing agents (e.g., educational agent being an appropriate instructional design) would create a perceived sense of positive energy (E), which would then stimulate the buoyancy of different psychological attributes (e.g., personal resolve). This process of optimization, in turn, would differ in terms of its magnitude (i.e., its strength: Phan and Ngu, 2019b), consequently as a result of the level of cognitive complexity of optimal best (e.g., Δ_(*L*1 –*L*2*A*)_ is not as difficult, cognitively, as Δ_(*L*1 –*L*2*B*)_, etc.). In this conceptualization, however, we propose that aside from optimization, a person’s future timespan (i.e., short vs. deep) would correspond to the cognitive level of optimal best – denoted as path “1,” path “2,” path “3,” etc. For example, the achievement of L_2*A*_ at T_2_, which is a relatively simple modeling, would perhaps be associated with a short future timespan.

Our first stipulation is that an extended FTP could coincide with and operate within the *process of optimization* in order to facilitate the achievement of optimal best. Optimization, extensively mentioned in the literature (e.g., Freund and Baltes, 1998; Fraillon, 2004; Ziegelmann and Lippke, 2007; Eguizábal et al., 2018), is an underlying process that serves to optimize an individual’s state of functioning (Phan and Ngu, 2017a). Drawing from Fraillon’s (2004) and Phan et al.’s (2017) seminal papers, we recently provided a comprehensive discussion of the *Framework of Achievement Bests*, which is a theoretical model that makes attempts to explain the process of optimization (Phan et al., 2019c). Optimization is like a “vehicle” that optimizes an individual’s state of functioning from one timepoint (e.g., T_1_) to that of another timepoint (e.g., T_2_). The Framework of Achievement Bests emphasizes two levels of best practice: (i) *realistic best*, or *actual best* (Fraillon, 2004), concerned with “a person’s current, realistic level of cognitive ability” (Phan et al., 2016) – for example, what is it that I am capable of at present in mathematics?; and (ii) *optimal best*, or *notional best* (Fraillon, 2004), concerned with “the maximum of a person’s cognitive capability” (Phan et al., 2016) – that is, what is my optimal best in mathematics? A notable inquiry arising from this focus is this: what *causes* and/or *facilitates* a person to progress from realistic best, L_1_, to optimal best, L_2_? Specifically, the totality of the process of optimization involves the active operation of three major pathways (see Figure 1):

i. *Pathway A*: the activation and enactment of different types of agents: educational (e.g., an instructional design for effective learning: Ngu et al., 2014), psychological (e.g., personal belief in efficacy: Bandura, 1997), and psychosocial (e.g., the importance of teacher-student social relationship: Roorda et al., 2011), which then act as sources of “energy.”
ii. *Pathway B*: the impact of energy on the stimulation of buoyancy of different types of psychological attributes – for example, intrinsic motivation, personal resolve, effective functioning, mental strength, and effort expenditure.
iii. *Pathway C*: the buoyancy of psychological attributes (e.g., mental strength) that may then arouse and sustain a person’s state of functioning and its improvement from T_1_ to T_2_.

According to Phan et al.’s (2019c) theorization, the combination of Pathway A, Pathway B, and Pathway C constitutes an “optimizing effect,” which the authors term “γ.” Aside from this positing of γ, the authors also proposed as an “Index of Optimization” (IO) where this equates with the following: Δ_(*L*2__–__*L*1)_ × γ, where Δ_(*L*2__–__*L*1)_ = quantitative difference between L_1_ and L_2_. Phan et al.’s (2019c) theoretical model of optimization, capitalizing on previous research developments (Fraillon, 2004; Phan et al., 2016, 2017, 2019a,c), is innovative for proposing a *quantitative nature* – that is, the possibility of measuring and quantifying the process of optimization. Furthermore, this consideration emphasizes the importance of the *magnitude of optimization* – that is, how “much” optimization is needed to facilitate the achievement of L_2_ from L_1_? This notion of magnitude, or strength, of optimization is interesting from our viewpoint since it places a focus on three major facets: (i) an individual’s state of motivation, personal experience, and level of L_1_; (ii) the quantitative and qualitative complexity of L_2_; and (iii) the difference or range between L_1_ and L_2_. Consider this example of L_1_ and L_2*A*_ where a secondary-school student is learning equation solving (Ngu and Phan, 2016):

• L_1_: the student knows how to solve one-step linear equations, for example: *x* + 10 = -4, solve for *x*.
• L_2*A*_: the student believes that he/she is able to solve multi-step linear equations, for example: 4 (*x* + 5) = 3 (*x* – 7), solve for *x*.
• L_2*B*_: the student believes that he/she is able to solve quadratic equations, for example: 4 (*x* + 8)^2^ = 6, solve for *x*.

As stated from the example, L_2*B*_ is more complex than L_2*A*_, which in turn, is more complex than L_1_. Hence, referring to our previous contention, it would require a greater magnitude of optimization to help in the achievement of L_2*B*_ from L_1_ (i.e., L_1_ → L_2*A*_) than of L_2*A*_ from L_1_ (i.e., L_1_ → L_2*B*_). As an example in school contexts, the magnitude of optimization (e.g., how much optimization is needed for me to understand something?) for understanding instructional material may involve a student’s reliance on and/or use of different instructional designs and/or pedagogical practices (Ngu and Yeung, 2012; Ngu et al., 2014). Comparative pedagogical practices (e.g., the use multimedia information such as a YouTube video vs. an in-depth text), in this case, may inform a student’s L_1_ to L_2_ differently.

In a similar vein, educationally, a native speaker of English may find it difficult to study and learn Chinese Mandarin as a foreign language. Hence, similar to the case of L_2*B*_, a student learning Chinese Mandarin as a foreign language would require significant optimization, especially if he/she does not have prior experience of Chinese, intrinsic motivation, etc. With reference to FTP, in this analysis, the student may be counseled to envisage a long future timespan (e.g., 2 years to provide adequate time) for successful accomplishment. A short future timespan (e.g., 2 months), by contrast, we think would not provide an adequate timeframe for the student to achieve optimal best in learning Chinese. By the same token, in terms of educational resources for the purpose of optimization, we would expect to find the student relying on multimedia presentations, personal one-on-one scaffolding, etc. Hence, as a point for consideration, let us consider two different scenarios.

• L_1_: the student has elementary knowledge of Chinese Mandarin, which enables him/her to have a basic conversation with another person (Note: in this case, for the sake of argument, assume that the student can read and write about 50 Chinese characters).
• L_2*A*_: the student believes that he/she is able to read and write at least 150 Chinese characters. At the same time, L_2*A*_ may also stipulate the combination of different Chinese characters to have new meanings.
• L_2*B*_: the student believes that he/she is able to read and write at least 300 Chinese characters, with some of these being sophisticated in nature (e.g., the character of 孝, xiào, which means filial piety). L_2*B*_ may also stipulate the combination of different Chinese characters to have new meanings.

Similar to our previous example of mathematics, progressing from L_1_ to L_2*A*_ is easier than achieving L_2*B*_ from L_1_ for Chinese Mandarin learning. Knowing how to read and write 300 Chinese characters, some of which are sophisticated in nature, would require much more time (e.g., a future timespan of 2 years for accomplishment) and a sustained effort at optimization (e.g., the reliance on many multimedia sources).

Optimization, in its totality, is positive in nature and reflects the paradigms of *positive psychology* (Seligman and Csíkszentmihályi, 2000; Seligman et al., 2009) and *motivation* (Franken, 2007). The enactment and/or the operational functioning of optimization is postulated to result in and to account for the achievement of optimal best, whereas inactive optimization is likely to give way to ineffective learning and sub-optimal achievements. Optimization, in this case, is more than just testament of an association between two variables (Phan et al., 2019c). A positive correlation (*r*), in this case, is somewhat limited and does not explain and/or reflect the full scope of the process of optimization. The proposition of an “optimizing effect,” denoted as γ, as Phan et al. (2019c) propose, is interesting and may depict and explain the complex nature of achievement of optimal best. Empirical research into the underlying mechanisms of optimization is somewhat limited to date with the exception of a few correlational inquiries that we have undertaken (e.g., Phan et al., 2018, 2019a,b, 2020; Phan and Ngu, 2019b). One possible reason for this limitation, we contend, is related to the “appropriateness” of a specific methodology that could assist the accurate measurement and assessment of optimization – we refer to this as “methodological appropriateness.”

### An Extended FTP and the Achievement of Optimal Best (FTP → OB)

How does an extended FTP assist in the achievement of an optimal level of best practice? In this analysis, we rationalize that a deep or long timespan into the future may act in tandem with, and/or form part of, the process of optimization (Fraillon, 2004; Phan and Ngu, 2017a; Phan et al., 2017, 2019c). Our conceptualization, illustrated in Figure 1, shows a person’s FTP acting as a psychological agent of change. This conceptualization, developed from our recent revision (Phan et al., 2019c) of the theory of optimization (Phan and Ngu, 2017a; Phan et al., 2017), shows the potential impact of FTP on the process of optimization.

As shown in Figure 1, we propose that a deep future timespan would correspond to the achievement of a complex level of optimal best – denoted in Figure 1, in this case, as path “1,” path “2,” and path “3.” With reference to this proposition, let us consider three different possibilities: (i) the achievement of L_2*A*_ from L_1_; (ii) the achievement of L_2*B*_ from L_1_; and (iii) the achievement of L_2*C*_ from L_1_. As depicted visually, the difference between L_1_ and L_2*C*_ (L_1_ – L_2*C*_) is “larger” than the difference between L_1_ and L_2*B*_ (L_1_ – L_2*B*_) and this difference, in turn, is larger than the difference between L_1_ and L_2*A*_ (L_1_ – L_2*A*_). Referring back to our previous discussion, the complexity of L_2*C*_ is greater than that of L_2*B*_ and L_2*A*_ (and the complexity of L_2*B*_ is greater than L_2*A*_). The achievement of L_2*C*_ therefore would require “more” time and optimization (i.e., the magnitude of optimization is relatively high). Our rationalization posits that envisaging and planning a deep future timespan would facilitate and encourage the striving of a more complex level of optimal achievement best. For example, a Year 11 student envisaging a timespan into the future about university life would, perhaps, strive to achieve more difficult and ambitious endeavors at present (e.g., obtaining consistent A and A^+^ grades for different subjects). Envisaging a shorter future timespan, from our point of view, would negate a student’s motivation and their desire to seek out complex endeavors for accomplishment (e.g., personal contentment with C and C^+^ grades for different subjects). As such, a relatively non-complex cognitive level of optimal best would require a lesser period of time for accomplishment.

As Simons et al. (2004b) explain, individuals with long or deep FTPs set goals that are situated in the distant future, whereas individuals with short FTPs set most of their goals in the near future (p. 122). Moreover, we would contend that an extended FTP is indicative of personal maturity, thoughtful deliberation, and well-measured ambitions. From this understanding, an investment in a long or deep FTP is noteworthy and in terms of academic pursuits, for example, a deep FTP could help students anticipate the following: (i) consider spending a longer period of time seeking assistance by consulting with others and/or utilizing different resources (e.g., going to the library); (ii) consider spending a longer period of time on personal reflection; and (iii) plan, organize, and develop a myriad of objectives and goals to assist in achieving a complex optimal best. A purposively extended FTP is more likely to direct a student’s attention, cognition, and motivation toward achieving specific complex optimal bests in life. Having a deep FTP is advantageous as this extended timespan (e.g., I need to spend the next 2 months studying this) would act as a source of energy, guiding the student’s behavior, personal resolution, and personal belief to recognize that anticipating long futures is beneficial and not wasteful.

We argue that conceptualization of an extended future timespan is not wasteful but may enable a student to consult others, engage in different types of cognitive strategies, and work on problems that could help improve his/her optimal level of best practice. As Lens et al.’s (2012) writing suggests, many of us have long FTPs whereas others, in contrast, may have relatively short FTPs. Those with short FTPs envisage and set most of their goals in the near future. They do not take into consideration what will come later on in life. Coupled with previous descriptions (Simons et al., 2004b; Zimbardo and Boyd, 2008; Lens et al., 2012), we contend that a short FTP is ineffective as this would negate the motivation, personal belief, and achievement of complex optimal best. In other words, a short FTP is intrinsically linked with a modest level of cognitive complexity. What this means then, of course, is that we do not expect a person with a short future timespan to achieve successfully a high level of cognitive complexity – for example, a relatively short future timespan of 2–3 weeks is unlikely, in this case, to be sufficient for preparing a student to plan, organize, and accomplish a complex level of optimal best. By contrast, however, a deep future timespan is more meaningful and advantageous as this would provide sufficient time (i.e., duration) for a person to reflect, contemplate, plan, seek help, etc (Zimbardo and Boyd, 2008; Husman et al., 2016).

Moreover, we contend that an extended future timespan is important as this duration provides adequate time to help facilitate and enact the process of optimization. In this sense, enactment of optimization is not instantaneous, but rather requires some timeframe for development, depending on the level of cognitive complexity of a given task. Something that is easy, for instance, would not require too much optimization – and hence, a shorter period of time in this case would be needed. Aside from this understanding, we also theorize that future timespan could fundamentally relate to a person’s *perceived sense of subjective task value* for learning (e.g., how important is this task?), and his/her *expectation to succeed* at the given task. In this analysis, with reference to this postulation, there are two considerations:

i. Positive task values (e.g., a student perceives that learning algebra is important for her/his future mathematics-related career plan), acting as a psychological agent, would motivate a person to persist and, likewise, to expend more effort into his/her studying and learning experiences (Eccles et al., 1983; Wigfield, 1994; Wigfield and Eccles, 2000). Valuing a particular task for significant personal reasons, we suggest, necessarily calls for an investment of time, planning, effort, resources, etc. In contrast, of course, a perceived minimal value of a learning task would not act as an optimizing agent of change.
ii. Expectation of achieving a complex optimal best would, likewise, act as a psychological agent in the process of optimization. A high level of expectation to succeed, which, in this case, a person is confident of achieving would energize and motivate the expenditure of time, effort, personal resolve, etc. A low level of expectation to achieve complex optimal best, by contrast, would deter the process of optimization.

Moreover, a perceived sense of subjective value and high expectation to succeed in the achievement of optimal best would, from our point of view, necessary require consideration of an appropriate future timespan. In this analysis, we would expect to find an association between a deep future timespan and a high level of subjective value and expectation – for example, a student who values Chinese Mandarin (i.e., perceives its importance for his/her future plans) and expects to succeed is likely, in this case, to recognize that a prolonged future timeframe would be needed to accomplish the task. A low level of expectation and the perception of low subjective value, by contrast, would not necessarily equate to a deep future timespan – in other words, a low level of expectation and low subjective value would instead correspond with a short future timespan. Time would be considered a minor factor as there would be a perception of its minimal value, reinforced by a low expectation of success and, hence, from the student’s point of view, envisaging a deep future timespan would be non-logical to the point of irrelevancy. Having said this, however, we also acknowledge the importance of cost-benefit factors (e.g., time spent vs. intensity – or anticipations of either or both) with reference to a person’s perceived subjective interests and well-being. Does envisaging a deep future timespan for the sake of achieving optimal best seem justified in all cases, especially when one considers the potential cost involved (e.g., the amount of effort, personal resolve, etc.)?

### Complexity of Optimal Best and Its Impact on an Extended FTP (OB → FTP)

How does optimal best in a subject matter assist in the development of a deep FTP? Unlike our first stipulation, we rationalize that in this case, a person’s striving to achieve a complex optimal level of best practice would construct a deep timespan into the future. As existing research inquiries have noted, we all differ in our FTPs – some of us have long and deep FTPs, while others have short FTPs. Moreover, those of us who have long FTPs tend to set most of our future goals in the distant future, whereas those with short FTPs set future goals in the near future (Simons et al., 2004b). A near future can be next month whereas, by contrast, a distant future may consist of a timespan that is 5–7 years from now.

Common-sense thinking about human nature, suggests, of course, that we all have different timespans. Why is it that some of us have long FTPs and not others? Motivation, academic capability, personal ethos and philosophical belief, and/or confidence, in this case, may account for individual variations in the setting of a particular timespan. A pervasive question is, how do we encourage students to develop and sustain deep FTPs? Chou, from our previous example, may envisage a future timespan of 5–7 years for the completion of his Ph.D. Another student, by contrast, may not have this ambition and instead project a future timespan of a couple of months maximum. This example of disparity in future timespans has implications for educators, stakeholders, and policymakers. In particular, let us explore the topical theme of optimal best (Fraillon, 2004; Martin, 2006; Phan et al., 2016) and consider how this feat could serve as a means to encourage and promote an extended FTP.

Achievement of optimal best is subjective and, indeed, reflects a person’s personal best (Fraillon, 2004; Martin, 2006; Liem et al., 2012; Phan et al., 2016). Optimal best in a academic subjects, for instance, is not static but may improve over time, as a result of cognitive maturity, increasing effort expenditure, in-depth understanding, and personal growth. For example, at present, a Year 8 student may indicate that her optimal best in “essay composition” is the capacity to write a scholarly 2000-word essay about the life of Erik Erikson. By the time this student is in first-year university, she would reflect and realize that her previous optimal best is now somewhat “low” in terms of cognitive capability. No doubt, at this stage of cognitive maturity, composing and writing a 2000-word essay about Erikson’s life would be perceived as being easy. As has been discussed previously (Phan and Ngu, 2017a), a person’s optimal best at the present time (i.e., L_2_) eventually becomes prior cognitive experience (i.e., L_1_) and forms part of his/her repertoire of knowledge. What this entails is that there is no definitive limit to a person’s optimal best. However, an “unrealistic” level of optimal best would not be conducive, giving rise to inaccurate results.

To date, to our knowledge, there has been very little study of the importance of timespan with reference to the added factor of a person’s optimal achievement best. This line of inquiry, for us, is innovative and stipulates the notion that cognitive complexity of optimal best could act as a catalyst to encourage and facilitate the setting of deep FTPs. We propose that for a high level of cognitive complexity of optimal best to develop requires a certain amount of time into the future for its development. In terms of schooling, for example, we can consider different levels of cognitive complexity (van Merriënboer et al., 2003; van Merriënboer and Sweller, 2005) – for example: (i) a Year 8 student, Thomas, striving to achieve a maximum-scoring football season; (ii) a Year 9 student, Melissa, seeking to overcome a health issue; or (iii) a group of Year 12 students wanting to enroll in a postgraduate medical degree program. Clearly, some tasks and activities are less complex and require only a modest range of focus, motivation, persistence, and effort expenditure, whereas others are more complex and demand much more time, effort, persistence, etc. A deep future timespan, we suggest, is healthy and as such, it is sufficient to encourage individuals to consider a more complex optimal best – for example, within the context of academic learning, a student may choose to strive to achieve an “A” grade for algebra (Phan et al., 2020).

Achieving optimal best is a personal endeavor. In school contexts, there is a difference between the problem of “4 (*x* + 5) = 3 (*x* – 7), solve for *x*” and the problem of “4 (*x* + 8)^2^ = 10, solve for *x*” for L_2_. Likewise, there is a difference between knowing how to compose a two-page essay and knowing how to write a 100-page thesis dissertation in Chinese Mandarin. The level of cognitive complexity of L_2_, as we explained, is dependent on a number of factors, such as a student’s current level of understanding and knowledge and their state of motivation. Our argument is that setting a complex optimal best is a desirable feat, as this could stimulate and encourage a student to envisage an extended timespan into the future. This rationalization is logical as more time and effort would be needed for a student to achieve a complex level of optimal best. How long would it take a person to solve the “Collatz Conjecture” problem? How much time does a senior citizen need in order to achieve and experience a state of self-actualization? Likewise, for an extremely knowledgeable student, how much time would he/she need to achieve a moderate level of cognitive complexity of optimal best? These questions, for us, emphasize the important need for a person *to contemplate and strive for complex optimal bests* in different academic subjects, given this feat would then form the premise for him/her to consider a correspondingly appropriate future timespan. In essence, this postulation considers the potential influence of the cognitive complexity of optimal best in facilitating a person’s projection of a future timespan.

Achieving optimal best, we contend, may also reflect a person’s perceived sense of subjective task value for the given task. Successful achievement of optimal best in an academic subject would highlight the perceived interest, appreciation, and/or placement of importance that the person has for the subject itself – for example, the student sees this learning as relevant for their future career plans. Some task that is relatively simple, by contrast, could instead indicate a non-meaningful subjective value and insignificant concern for the subject matter. Indeed, a relatively simple task would not hold any significance and, more importantly, a person would not have much expectation about its importance. This consideration differs somewhat from the discussion in the preceding section, where we argued that the perceived subjective task value and high expectation would act as psychological agents (Phan et al., 2017, 2019c) in the optimization of achievement best. Here, in this section, we postulate that cognitive complexity of optimal best could influence the perception of the relevance, significance, and/or importance of a learning task. Something that is perceived as being difficult for optimal achievement is likely to instill a strong sense of belief in its subjective value. In the context of schooling, for example, the creation of high cognitive complexity in a topical theme for optimal best achievement would convey to students the message that the subject matter itself is of value. Such discourse (i.e., cognitive complexity of optimal best → value), in turn, would connote a specific future timespan for consideration. In contrast, of course, cognitive simplicity of the subject matter would indicate subjective perceptions of unimportance and insignificance.

### In Summary

**FIGURE 2:**
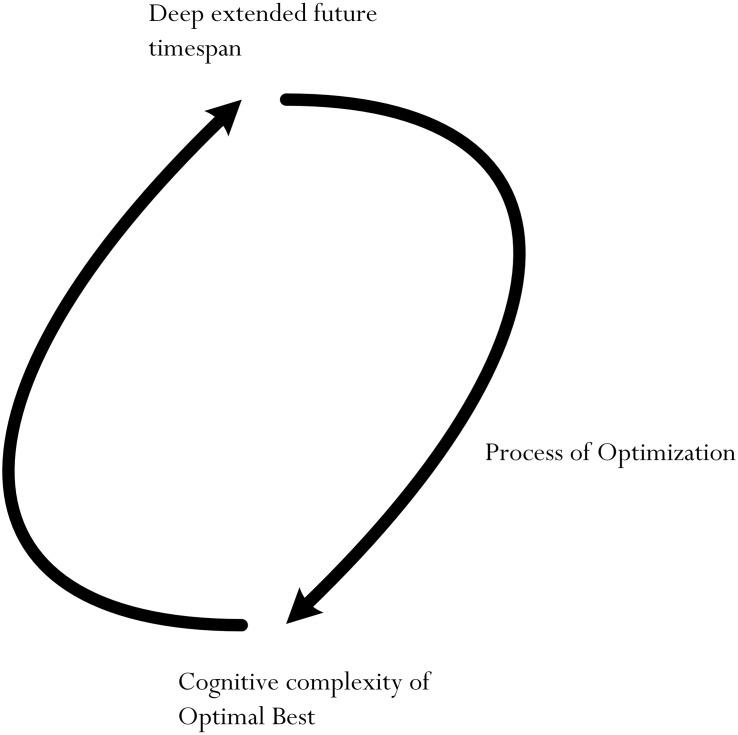
Cyclic relationship between extended timespan and cognitive complexity of optimal best.

The preceding sections have provided theoretical and conceptual accounts of the interrelationship between FTP and optimal achievement best. Our conceptualization, we contend, indicates a potential cyclic system, as shown in Figure 2. This cyclical system depicts the potential effect of a complex level of optimal achievement best on a deep extended future timespan and, in turn, this deep extended future timespan is observed as positively influencing the complex level of optimal achievement best. What does this conceptualization depict? We can consider the following:

• A deep timespan into the future is advantageous and beneficial, providing subjective grounding and opportunities for the successful enactment of optimization in order to facilitate in the striving of complex optimal bests (i.e., deep extended future timespan → complex optimal best).
• Achieving complex optimal bests requires adequate effort and time and, hence, a deep timespan into the future for a person to envisage what would be needed (i.e., complex optimal best → deep extended future timespan).

The research into FTP is clear and consistent (Lewin, 1951; Nuttin, 1964; Gjesme, 1983; de Bilde et al., 2011; Taylor and Wilson, 2019), while the study of the nature of optimal achievement best is still progressing (e.g., Phan et al., 2018, 2019a,b). One notable line of inquiry regarding optimal achievement that is of interest, at present, is related to its methodological account (Phan et al., 2019c) – for example, how do we measure, assess, and evaluate the true nature of optimal best? To do so our research has established a conceptualization that attempts to integrate two independent research inquiries into one holistic model. This consideration is innovative as it places emphasis on the positive interrelation of the potential for achieving optimal achievement best and extended future timespans. Arising from our description is a desirable proposition for development: *a high level of cognitive complexity of optimal best* and *a deep, meaningful future timespan* (Figure 2).

## Conclusion and Future Directions

Time is a mysterious concept. We cannot go into the past and change our past behaviors, feelings, experiences, etc. What we can do, though, is reflect upon and use past experiences to inform the present moment and set future goals for accomplishment. The future is unknown and, in many cases, we will never truly know what the future holds. Motivational research into the proactivity of human agency has led to extensive research inquiries emphasizing the interconnections between a person’s time experiences and his/her performance outcomes, academically and non-academically. Future time perspective is an interesting psychological concept for study, given that the future is always unknown and uncertain. One interesting aspect for development, in particular, is related to the promotion of the projection of deep, meaningful future timespans. Deep FTPs are healthy as they facilitate a heightened state of personal resolve, motivation, and effort expenditure.

It is not always easy for a person to spend time anticipating his/her future. Many of us, in this case, are content with our lives in the present moment. Thinking about future trajectories often appears to people as being somewhat meaningless, especially in light of day-to-day work demands and personal commitments, etc. By the same token, of course, it is uncertain whether a person’s anticipation of future goals, aspirations, etc., would be accurate or realistic enough to have a credible meaning. Despite this plausible criticism, we have proposed from an educational point of view that a focus on extended future time goals is a useful point for encouragement. We usually have to make plans anyway (e.g., “What I would like to do in the next 12 months”), organize our time schedules, work through our finances, etc. On this basis, anticipation of a positive future should be encouraged as this would direct a person’s focus and commitment toward something that is tangible. Accordingly, we need to consider the stipulation of an appropriate timespan. How “much” time into the future should one think about, anticipate, and/or project? As existing research (e.g., Simons et al., 2004a, b; Zimbardo and Boyd, 2008) has shown, an extended FTP is more healthy and robust than a short FTP, as this directs and motivates a person to work harder toward achieving his/her future goals. At the same time, a deep FTP may reveal and reflect a person’s state of seriousness, degree of commitment, and ambition. Having said this, of course, we also raise the issue whether a deep extended FTP could give rise to negative outcomes. How much time into the future should one anticipate before that timespan causes problems?

Our discussion has provided an in-depth examination of how FTP could relate to the nature of optimal achievement best. Specifically, within the context of educational application, our proposition emphasizes two major interrelated paths for further development: (i) whether variations in a person’s future timespan and, more importantly, increasing temporal distances of FTP (say, from 5 months to 5 years) motivate or detract from the achievement of cognitively complex optimal bests; and (ii) whether the types of complex optimal bests found in different academic subjects necessarily impact upon the extent of projection of FTPs. This proposal for a line of inquiry is both conceptual and philosophical, but also, we feel, requires empirical data to validate and affirm its value. What we have established so far is a preliminary inquiry; theoretically derived from existing independent research in the areas of FTP and optimal achievement best. There is a need for researchers to develop appropriate methodological designs that could measure, assess, and validate these proposed lines of inquiry (the nature of the relationships: Extended FTP → Complex OB and Complex OB → Extended FTP). For example, in relation to our proposed conceptualization, we suggest it would be worthwhile for researchers to develop different types of cognitive tasks, learning activities, etc., and to explore how these associate with a person’s projection of a future timespan. Is there a direct correlation, which could/would then result in a statistically-derived equation, pattern, etc., for general application?

**FIGURE 3:**
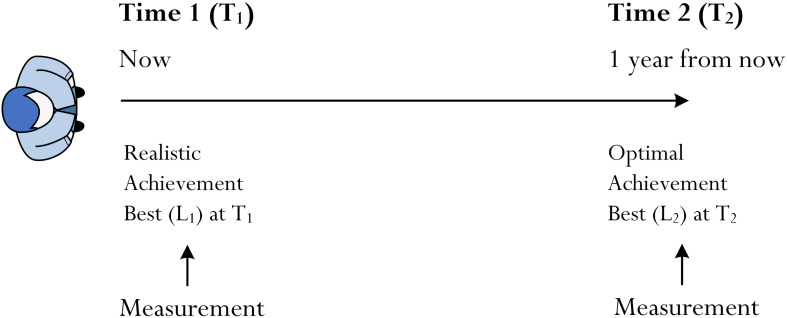
Proposed methodological design.

When we refer to time then, methodologically, research in this area necessarily would rely upon the collection of longitudinal data. An extended FTP places emphasis on a time duration or a time period into the future and, as such, it is sufficient to say that there is a “time difference,” denoted as Δ_(*T*1–*T*2)_, between now (T_1_), the present moment, and a particular point into the future (T_2_). It also needs to be kept in mind that the difference between T_1_ and T_2_ would not necessarily reflect either regular and constant development or at some time-point become instantaneous. Figure 3 summarizes our description and shows a simple methodological design, which consists of the collection of data on two separate occasions, T_1_ and T_2_. As an aside, it can be noted that in terms of the issues raised in this paper the difference between T_1_ and T_2_ is, indeed, analogous with an extended FTP. A positive quantitative difference between T_1_ and T_2_, we contend, should be reflect a realistic human-centered timespan (e.g., 1 year from now). What does this methodological proposal mean in terms of optimal achievement best?

The current authors recently published a detailed theoretical account of the process of optimization, where we specifically delved into the issues of methodology – i.e., how do we measure and assess optimization (Phan et al., 2019c)? One recommendation that we proposed included the use of the same measure of best practice (e.g., cognitive competence test) on multiple occasions (e.g., CCT-T_1_ and CCT-T_2_, where CCT = cognitive competence test) in order to provide a proxy indicator of the enactment of optimization. From Figure 3, we now propose that in order to attain an understanding and affirmation of the actual achievement of a goal at T_2_ (e.g., achieving optimal best in the topic of algebra), we would need to collect data on two occasions – say, now, T_1_, and 1 year from now, T_2_. We contend that it is inadequate and somewhat limited, in this case, simply to measure and collect data at T_1_. In other words, a measurement of achievement at the conclusion of an extended timespan (e.g., the achievement of a future goal at T_2_), alone, is limited as we would not know what actually occurred at T_1_ that may have led to achievement at T_2_. In a similar but converse vein, as we argued in our recent article (Phan et al., 2019c), a true indication of a person’s optimal achievement best still would require its measure at T_2_ and not just at T_1_.

Validating the cyclic system shown in Figure 2 would require the use of multi-wave panel data (Marsh and Yeung, 1997, 1998). This longitudinal methodological design could provide a grounding for researchers to establish the following patterns, say: T_1_ extended future timespan → T_2_ optimal achievement best; and T_1_ optimal achievement best → T_2_ extended future timespan (note: “→” = prediction). Evidence obtained could establish a cause-and-effect model for further experimental manipulation (Phan and Ngu, 2017a). In a similar vein, personal commitment, or a lack thereof, and what this means for a person’s subsequent achievement and fulfillment of future goals is a correlative line of inquiry. This line of inquiry is interesting and was raised by one of our reviewers for consideration. Indeed, personal commitment may give rise to a student’s personal resolve to strive for both short-term and long-term successes (Phan et al., 2020).

We acknowledge from the literature that having a deep future timespan is valuable, as this instills a sense of motivation and guides and directs a person’s cognition and behavior toward the future goal(s) (e.g., a student’s striving to enroll in medical school). At the same time, of course, a deep future timespan may reflect a person’s hopes and ambitions, as well as his/her mental fortitude, to achieve different types of long-term goals. Having said this, however, we do have some reservations, which we previously described, regarding the “depth” of future timespan (e.g., 5 years in duration). For example, in the context of schooling, how does a student remain autonomous, independent, and/or disciplined enough to sustain his/her state of motivation, and/or to remain on task over a 3-year period? Uncertainties, personal circumstances, and extraneous influences may, individually and/or in combination, act to derail, negate and demotivate a student from maintaining his/her state of motivation and discipline to remain on task. When this is the case, we would not necessarily expect the student to achieve and fulfill his/her future goals. On this basis, what would educators and/or researchers have to understand to counter the problem of sustaining motivation, focus, discipline, etc.?

In a recent study, Oyama et al. (2018) proposed a term known as the “Hemingway effect,” which is defined as “a positive effect of not completing a task” (p. 8). According to the authors’ rationalization, “there are certain conditions, [when] not completing a task can actually enhance people’s motivation to engage in the task – to complete or continue it” (p. 8). The *Hemingway effect*, we contend, may associate with our previous concern regarding the sustaining and continuation of a student’s motivation and discipline to achieve future long-term goals. In this analysis, we postulate that the Hemingway effect could act to guide a student’s state of cognition, motivation, and/or behavior over the course of time. The results of Oyama et al.’s (2018) study, interestingly, indicate that there are two conditions by which the Hemingway effect would occur: (i) explaining and clarifying for a student what is needed to complete the unfinished task(s); and (ii) a student’s perception of closeness to completing that task. It would be of interest for researchers to consider the potential of the Hemingway effect to guide the way students and educators might envisage a deep future timespan to achieve optimal best. In other words, researchers could explore whether and/or to what extent a student’s perception of closeness to completing a task helps to guide his/her focus of attention and discipline, as well as sustaining his/her state of motivation.

Finally, in tandem with Phan et al.’s (2019c) recent publication, we contend that the issue of measurement and assessment of optimal best practice is currently somewhat inconclusive. Most particularly, how does an educator measure and/or determine the complexity of a person’s optimal best? This question is even more poignant when we incorporate the complicating factor of future timespans. Most particularly in this case, how would we determine the extent of accuracy or usefulness of a person’s projection of his/her future timespan? In a similar vein and the subject at the core of this article, how could we accurately measure that the complexity of optimal best is aligned with a person’s consideration of deep future timespan – that is, does achieving more complex levels of optimal best require more time, as suggested by our proposition, while achieving less complex levels of optimal best require shorter time periods? As have been discussed before, it is also of interest to consider the potential association between the cognitive complexity of optimal best and future timespan with reference to a person’s determination and/or perceived sense of task-value for learning – in particular, the quest for researchers to design and develop a robust methodological design that could measure and assess a person’s determination and/or perceived sense of task-value when optimal best is achieved and/or when a specific future timespan is set.

## Author Contributions

HP and BN contributed equally to the conceptualization, articulation, and write up of this manuscript. KM contributed to the revision of this manuscript.

## Conflict of Interest

The authors declare that the research was conducted in the absence of any commercial or financial relationships that could be construed as a potential conflict of interest.
